# Metformin mediates neuroprotection and attenuates hearing loss in experimental pneumococcal meningitis

**DOI:** 10.1186/s12974-019-1549-6

**Published:** 2019-07-27

**Authors:** Lukas Muri, Ngoc Dung Le, Jonas Zemp, Denis Grandgirard, Stephen L. Leib

**Affiliations:** 1 Neuroinfection Laboratory, Institute for Infectious Diseases, University of Bern, Friedbühlstrasse 51, 3010 Bern, Switzerland; 2 Graduate School for Cellular and Biomedical Sciences (GCB), University of Bern, Mittelstrasse 43, 3012 Bern, Switzerland

**Keywords:** Pneumococcal meningitis, Brain injury, Neuroinflammation, Neurologic sequelae, Metformin, Inner ear damage, Neuroregeneration

## Abstract

**Background:**

Pneumococcal meningitis is associated with high risk of neurological sequelae such as cognitive impairment and hearing loss. These sequelae are due to parenchymal brain and inner ear damage primarily induced by the excessive inflammatory reaction in response to bacterial brain invasion. Metformin—a biguanide drug to treat diabetes mellitus type 2—was recently found to suppress neuroinflammation and induce neuroregeneration. This study evaluated the effect of metformin adjunctive to antibiotics on neuroinflammation, brain and inner ear damage, and neurofunctional outcome in experimental pediatric pneumococcal meningitis.

**Methods:**

Eleven-day-old Wistar rats were infected intracisternally with 5.22 ± 1.27 × 10^3^ CFU *Streptococcus pneumoniae* and randomized for treatment with metformin (50 mg/kg, i.p., once daily for 3 weeks) plus ceftriaxone (100 mg/kg, i.p., bid, *n* = 61) or ceftriaxone monotherapy (*n* = 79). Cortical damage and hippocampal apoptosis were evaluated histomorphometrically 42 h post infection. Cerebrospinal fluid cytokine levels were analyzed during acute infection. Five weeks post infection, auditory brainstem responses were measured to determine hearing thresholds. Spiral ganglion neuron density and abundance of recently proliferated and integrated hippocampal granule neurons were assessed histologically. Additionally, the anti-inflammatory effect of metformin was studied in primary rat astroglial cells in vitro.

**Results:**

Upon pneumococcal infection, metformin treatment significantly reduced levels of inflammatory cytokines and nitric oxide production in cerebrospinal fluid and in astroglial cell cultures in vitro (*p* < 0.05). Compared to animals receiving ceftriaxone monotherapy, adjunctive metformin significantly reduced cortical necrosis (*p* < 0.02) during acute infection and improved median click-induced hearing thresholds (60 dB vs. 100 dB, *p* < 0.002) 5 weeks after infection. Adjuvant metformin significantly improved pure tone hearing thresholds at all assessed frequencies compared to ceftriaxone monotherapy (*p* < 0.05) and protected from PM-induced spiral ganglion neuron loss in the inner ear (*p* < 0.05).

**Conclusion:**

Adjuvant metformin reduces brain injury during pneumococcal meningitis by decreasing the excessive neuroinflammatory response. Furthermore, it protects spiral ganglion neurons in the inner ear and improves hearing impairments after experimental pneumococcal meningitis. These results identify adjuvant metformin as a promising therapeutic option to improve the outcome after pediatric pneumococcal meningitis.

**Electronic supplementary material:**

The online version of this article (10.1186/s12974-019-1549-6) contains supplementary material, which is available to authorized users.

## Introduction

*Streptococcus pneumoniae* and *Neisseria meningitidis* are the most prevalent pathogens of childhood meningitis beyond neonatal age [1, 2]. Pneumococcal meningitis (PM) is associated with a high risk for neurologic disabilities, as one-third of PM survivors present with neurofunctional sequelae including hearing loss, epilepsy, cerebral palsy, cognitive impairment, and learning disability [3–5]. Pneumococci are reported to reach the brain through the bloodstream after successful nasopharyngeal colonization with subsequent bloodstream invasion and survival [4, 6]. Bacteria are proposed to transmigrate through the blood-brain barrier (BBB) by receptor-mediated endothelial adhesion and transcytosis using an interplay between the platelet-activated factor receptor (PAFr), polymeric Ig receptor (pIgR), and platelet endothelial adhesion molecule (PECAM-1) expressed on the host’s endothelial cells [6–11]. Notably, pneumococci might also gain access to the CNS through a paracellular route by disrupting the BBB’s integrity via pneumolysin- and α-glycerophosphate oxidase (GlpO)-induced damage to endothelial cells [12–14]. In addition, pneumococcal CNS infections may arise after local spreading of bacteria from nearby focal infections (i.e., otitis media, sinusitis, or mastoiditis). Upon recognition of the invading pathogens, recruited neutrophils together with brain-resident immune cells produce high levels of inflammatory cytokines, reactive oxygen and nitrogen species (ROS and RNS), intended to eliminate the invading pathogens but also causing collateral damage to the vulnerable brain tissue [4, 15, 16]. This excessive neuroinflammatory response upon pneumococcal cerebrospinal fluid (CSF) invasion together with pneumococcal toxins contributes to PM-associated central and peripheral nervous system (CNS and PNS) injury [4, 17–20]. A hallmark of PM is the intense neutrophilic pleocytosis in the CSF in reaction to invading bacteria [21–23]. PM-induced brain injury is characterized by cortical necrosis—as a consequence of ischemia and hypoxia by a combination of reduction blood perfusion of the brain with focal cerebral vasculitis and vasospasms [24, 25]. Apoptosis in the hippocampal dentate gyrus involving granular cell progenitors [26–29]—which has been linked to learning and memory impairment in independent experimental models [30–32]—represents a second form of neuronal damage upon PM. Furthermore, spreading pneumococci and leukocytes from the CSF to the perilymphatic space of the cochlea result in damage of cochlear sensory hair cells and spiral ganglion neurons (SGNs) in the inner ear [5, 33–35], being responsible for hearing loss in up to 30% of surviving patients [3, 36, 37].

Corticosteroids are recommended as adjuvant therapy to downmodulate the detrimental inflammation during PM [38, 39]. In a comprehensive meta-analysis, dexamethasone was shown to improve the outcome of adults with PM and children with meningitis caused by *Haemophilus influenzae* type b—without showing a beneficial effect in pediatric PM [38]. In experimental pediatric PM, dexamethasone even aggravates hippocampal regeneration and learning deficiency [32, 40]. Therefore, it is important to find alternative therapeutic approaches to alleviate the diseases burden in children suffering from PM.

Metformin is a biguanide drug widely used for the treatment of patients with type 2 diabetes since the 1960s. It enhances insulin sensitivity, induces glycolysis, and suppresses gluconeogenesis in the liver [41]. Metformin activates the adenosine monophosphate-activated protein kinase (AMPK) in hepatocytes, resulting in the downregulation of the expression of genes involved in hepatic gluconeogenesis [42]. Important in the framework of brain inflammation is the finding that AMPK activation suppresses the expression of pro-inflammatory cytokines in lipopolysaccharide (LPS)-injected rats [43]. In experimental multiple sclerosis, metformin significantly reduced neuroinflammation in vitro and in vivo [44]. Metformin reduced neuroinflammation and hippocampal cell death in diabetic mice, with subsequent improved performance in spatial memory tests [45] and was shown to be neuroprotective in experimental Parkinson’s disease [46]. Furthermore, metformin protected CA1 pyramidal cell of the hippocampus from ischemia/reperfusion injury [47] and protected cochlear hair cells from gentamicin-induced toxicity [48]. In addition to its neuroprotective effects, metformin has recently been shown to potently promote neurogenesis in the hippocampus and cortex by enhancing neural precursor self-renewal, proliferation, and differentiation [42, 49]. Increased neurogenesis upon metformin treatment resulted in improved memory formation in multiple experimental models of brain injury [47, 50–53].

We therefore hypothesize that adjuvant metformin during acute PM reduces the excessive neuroinflammation and subsequent brain and cochlear injury—hallmarks of poor prognosis in PM. In addition, chronic metformin application may support neuroregeneration, thereby improving neurofunctional outcomes after PM.

## Methods

### Infecting organism

A clinical isolate of *Streptococcus pneumoniae* (serotype 3) from a patient with bacterial meningitis was cultured overnight in brain heart infusion (BHI) medium, diluted tenfold in fresh, pre-warmed BHI medium and grown for 5 h to reach the logarithmic growth phase. The bacteria were centrifuged for 10 min at 3100×*g* at 4 °C, washed twice, resuspended in saline (NaCl 0.85%), and further diluted in saline to the desired optical density (OD_570nm_). The inoculum concentration was determined by serial dilution and culturing on Columbia sheep blood agar (CSBA) plates. Bacterial preparation for *S*. *pneumoniae* D39 wild type (serotype 2) and its non-encapsulated mutant R6 were performed as described above with minor adaptions. Overnight cultivation was shortened to 8 h and time for logarithmic growth was reduced to 1.5 h for D39.

### In vitro astroglial cells stimulation

Astroglial cells consisting of astrocytes, microglia, and oligodendrocytes were isolated from infant rat brains at postnatal day 3 (P3), as previously reported [54]. Briefly, rats were sacrificed by decapitation and brains were isolated. The cortices were homogenized mechanically in PBS by pipetting up and down with a 5 ml plastic pipette, centrifuged (500×*g*, 7 min, 4 °C) and resuspended in DMEM (Sigma-Aldrich, Merck Switzerland) containing 5% FCS (Biochrom, Germany), GlutaMAX™ (ThermoFisher, Switzerland), and antibiotic-antimycotic solution (ThermoFisher, Switzerland). After resuspension, cells were plated in T75 flask (TPP®, Merck) previously coated with poly-l-ornithine (PLO, 0.01 mg/ml in PBS, Sigma-Aldrich). On day 11 post isolation, cells were seeded on PLO-coated 24-well plates, at a density of 200,000 cells/well. Astroglial cells were stimulated with (a) 10 μg/ml lipopolysaccharide (from *Escherichia coli*, L2654, Sigma-Aldrich) in PBS, (b) heat-inactivated *S*. *pneumoniae* serotype 3 (3 × 10^7^ CFU/ml), or (c) living *S*. *pneumoniae* serotype 3 (2 × 10^7^ CFU/ml) treated with ceftriaxone (CRO, 12 mg/ml, Rocephine, Roche). For each stimulus, cells treated with metformin (metformin hydrochloride, 10 mM, Sigma-Aldrich) were compared to vehicle-treated cells. Heat-inactivation of *S*. *pneumoniae* serotype 3 was performed in a thermo-shaker (Thermomixer comfort, Vaudaux-Eppendorf AG, Switzerland) at 56 °C for 30 min. To confirm serotype-independent anti-inflammatory properties of metformin, its effect was further assessed on astroglial cells stimulated with living D39 (6 × 10^7^ CFU/ml) or R6 (7 × 10^7^ CFU/ml) treated with ceftriaxone. The use of ceftriaxone after stimulation with living bacteria is required to guarantee astroglial cell survival. Additionally, it mimics the in vivo model with antibiotic-induced bacteriolysis, which initially increases the neuroinflammatory reaction upon antibiotic treatment initiation.

### Quantification of nitric oxide production in vitro

After 24 h of astroglial cell stimulation, 100 μl of the cell culture supernatant from each well was collected and centrifuged (13,000 rpm, 5 min, 4 °C), transferred to a 96-well plate, followed by addition of 100 μl Griess reagent (Sigma-Aldrich). NO_2_^−^ content was determined by measuring the absorbance at 550 nm with a microplate reader (Molecular Devices, THERMO max). A serial dilution of NaNO_2_ solution (Sigma-Aldrich) from 100 to 1.5625 μM was measured and used to create a standard curve for quantification. NO_2_^−^ concentration in cell culture supernatant served as a proxy for NO release, and is further addressed as NO.

### Infant rat model of pneumococcal meningitis

All animal studies were approved by the Animal Care and Experimentation Committee of the Canton of Bern, Switzerland (license no. BE 129/14 and 01/18). A well-established infant rat model of pneumococcal meningitis was used for this study [35, 55]. Eleven-day-old female and male Wistar rat pups and their dams were purchased from Charles Rivers (Sulzfeld, Germany). The dams were provided with tap water and pellet diet ad libitum. Litters were kept in rooms at a controlled temperature of 22 ± 2 °C. During the acute phase of the disease (from infection to 42 h after infection), animals were housed in a room with natural light. For the long-term experiments, the animals were transferred to individually ventilated cages (IVC) in a room with controlled 12 h light/dark cycles. The pups were infected intracisternally with 10 μl of the inoculum containing 5.22 ± 1.27 × 10^5^ CFU/ml of living *S*. *pneumonia* serotype 3. Control animals were injected with 10 μl saline. Meningitis was confirmed by quantitative analysis of bacterial titers in the cerebrospinal fluid (CSF) at 18 hpi, where 5 μl of CSF were collected by puncture of the cisterna magna, followed by serial dilution and cultivation on CSBA plates.

A total of 174 infant were included in this study, representing 17 independent experiments with 7, 12, or 14 infant rats per experiment. Seventy-six animals were investigated during acute PM to assess neuroinflammation and brain damage, and 98 animals were studied to investigate neurofunctional outcomes. Infected and mock-infected animals were randomized for treatment with adjuvant metformin (50 mg/kg, i.p.) and/or CRO (100 mg/kg, i.p., twice daily [b.i.d.]). Animals with CRO monotherapy received a i.p. vehicle injection. All animals received the same amount of fluids during the experiment. Both therapies were initiated at 18 hpi. For the acute experiments, clinical scoring was performed by investigators blinded to treatment modalities of individual animals. The animals were weighted and clinically scored according to the following scoring scheme (1 = coma, 2 = does not turn upright, 3 = turns upright in > 5 s, 4 = turns upright in < 5 s, 5 = normal) at 0, 18, 24, and before sacrificing at 42 hpi. Spontaneous mortality was documented. CSF samples at 18, 24, and 42 hpi were obtained by puncture of the cisterna magna using a 30-gauge needle. CSF samples were centrifuged (16,000×*g*, 10 min, 4 °C) and supernatants were stored at − 80 °C for later CSF cytokine measurements. For long-term experiments, CRO therapy was continued for the first 5 days after infection. Animals treated with metformin received chronic daily metformin application for additional 2 weeks, whereas animals from the CRO monotherapy group received daily vehicle injections. In addition, bromodeoxyuridine (BrdU, 50 mg/kg, Sigma-Aldrich) was given once daily on day 3–5 after infection combined with CRO. Animals were sacrificed with pentobarbital (Esconarkon®, 150 mg/kg, i.p., Streuli Pharma AG, Switzerland) and perfused with 4% paraformaldehyde (PFA, Merck) in phosphate-buffered saline (PBS). Brains and cochleas were harvested and fixed in 4% for histological analysis.

### Histomorphometric analysis of cortical damage and hippocampal apoptosis

Damage to the cerebral structures was quantified in all animals sacrificed at 42 hpi, as previously described by us and other independent research groups [56–59]. Brains were fixed in 4% PFA and cryopreserved in 18% sucrose in PBS at 4 °C overnight. Brain cryosections (45 μm) were systematically sampled with a cutting frequency of 15 and stained for Nissl substance with cresyl violet. Cortical damage was defined as areas of decreased neuronal density with histological features of necrosis, using ImageJ software. Dead cells with histological features of apoptosis were quantified in 48 visual fields spanning the hippocampal dentate gyrus by using × 400 magnification. Histologic assessment was performed and evaluated by investigators blinded to treatment modalities of the individual animals.

### Analysis of cytokine expression in CSF

Cytokines known to be upregulated during PM (IL-1β, IL-6, TNF-α, IL-10, and IFN-γ) were assessed using magnetic multiplex assay (Rat Magnetic Luminex® Assay, Rat Premixed Multi-Analyte Kit, R&D Systems, Bio-Techne) on a Bio-Plex 200 station (Bio-Rad Laboratories) as described previously [35, 60]. Five microliter of CSF harvested at 18, 24, and 42 hpi were diluted to a final volume of 50 μl. For in vitro samples, 50 μl of cell culture medium was used undiluted. For each sample, a minimum of 50 beads was measured. If the concentration of the sample was below the detection limit, a value corresponding to the detection limit provided by the manufacturer was used, considering the dilution factor. The detection limits for undiluted samples were 2.93 pg/ml for IL-1β, 23.2 pg/ml for IL-6, 8.95 pg/ml for IL-10, 11.5 pg/ml for TNF-α, and 70.9 pg/ml for IFN-γ.

### Assessment of hearing capacity by measuring auditory brainstem response

Five weeks post infection, auditory brainstem responses (ABR) were performed to determine the hearing thresholds of the animals, as described previously [35, 60]. Click and pure tone recordings were performed with the Intelligent Hearing Systems SmartEP system. Animals were anesthetized with 5% isoflurane (Attane TM, Piramal healthcare). The anesthetized animal was placed on a heating pad in a soundproof chamber. A rubber facial mask was put on the animal for continuous delivery of isoflurane. As soon as the animal was under deep anesthesia, the isoflurane concentration was reduced to 2%. Subdermal electrodes were placed in the mastoid of the tested ear (active), at the vertex (reference) and in the lower limb (ground). Earphones were placed in the outer auricular canal. Click stimuli and 5-ms pure tone bursts (4, 8, 16, and 32 kHz) were presented at a rate of 21.1 s^−1^, ranging from 100 to 20 dB sound pressure level (SPL) in 10 dB decrements (5 dB decrements close to threshold). The hearing threshold was defined as the lowest intensity that induced a visually detectable first peak. Hearing thresholds were independently analyzed by investigators blinded to treatment modality.

### Immunohistological assessment of spiral ganglion neuron density

Six weeks post infection, the region of the temporal bone containing the cochlea was dissected from skull, fixed in 4% PFA, and then transferred to OsteoSoft® (Merck) for 7–10 days. OsteoSoft was replaced by 30% sucrose in PBS 24 h before cutting. Left cochleas were used for the spiral ganglion histology, whereas the right ones were kept as back-up. Then, 14 μm sections were cut and mounted on Superfrost Plus Menzel glass slides. The staining procedure was performed with a Shandon Sequenza staining rack (Thermo Fisher Scientific). Sections were permeabilized for 5 min with 0.1% Triton-X, followed by blocking with blocking solution (PBS with 2% BSA and 0.01% Triton-X) for 1 h at room temperature (RT). The primary antibody βIII-tubulin (1:500, mouse-anti-rat, Promega G712A) was diluted in blocking solution and incubated overnight at 4 °C. The sections were rinsed with 3 × 500 μl PBS and incubated with the secondary antibody (goat-anti-mouse Alexa Fluor 488, 1:500, Thermo Fisher Scientific, A-11029) for 2 h at RT. After rinsing again, the slides were mounted with Fluoroshield™ with DAPI (Sigma-Aldrich). Images of the spiral ganglions were acquired using a fluorescent microscope (ZEISS Axio, Imager M1, West Germany) equipped with a digital camera (Zeiss AxioCam HRc).

### Immunohistological assessment of BrdU^+^ and NeuN^+^ cells in the hippocampal dentate gyrus

Brains were harvested 6 weeks post infection and fixed in 4% PFA for 4 h and stored in PBS at 4 °C for later embedding in paraffin. Ten micrometer sections were cut using a microtome (Microm, Germany). Every 18th section was sampled on Superfrost Plus Menzel glass slides and air dried. Sections were deparaffinized and submitted to antigen retrieval by incubating the slides in sodium citrate (Merck KGaA) 10 mM pH 6.0 for 1 h in a 95 °C water bath. The brain sections were permeabilized and blocked as described above. The primary antibodies BrdU (1:500, sheep-anti-rat, abcam, ab1893) and NeuN (1:500, mouse-anti-rat, Millipore, MAB377) were diluted in blocking solution, added to the slides and incubated overnight at 4 °C. The slides were washed 3 × 5 min with PBS and the secondary antibodies—donkey-anti-sheep Alexa Fluor 488 (1:500, Thermo Fisher Scientific, A11015) and donkey-anti-mouse Cy3 (1:500, Jackson ImmunoResearch, 715-165-151)—were added for 2 h at RT. After washing the sections 3 × 5 min in PBS, they were mounted with Fluoroshield containing DAPI and kept at 4 °C in dark until imaging. BrdU^+^ cells in the NeuN^+^ region of the dentate gyrus were quantified using a × 400 magnification on fluorescent microscope. The first six sampled sections containing the dentate gyrus with the upper and lower blades connected were quantified. A mosaic picture of each dentate gyrus was created with Zeiss AxioVision software using individual pictures taken with the DAPI fluorescence channel at × 100 magnification. The mosaic was used for determining the area of the dentate gyrus with ImageJ software. Number of BrdU^+^ cells were then calculated and evaluated as number of BrdU^+^ cells per mm^2^ dentate gyrus granule cell layer.

### Statistical analysis

Statistical analyses were performed with GraphPad Prism (Prism 7; GraphPad Software Inc., San Diego, USA). Results are presented as mean values ± standard deviation if not stated otherwise. Survival was calculated using a log rank (Mantel-Cox) test. To compare differences between means of two normally distributed groups, an unpaired Student *t* test or Welch’s *t* test were used (cortical damage assessed with Welch’s *t* test). To assess differences between non-normal distributed data (hearing thresholds, hippocampal apoptosis), a non-parametric Mann-Whitney test was used. A two-way ANOVA was performed to analyze differences between treatment modalities and to pure-tone hearing frequencies. Repeated-measure ANOVA was used to assess differences in spiral ganglion neuron density from the three cochlear regions (base, middle, apex). Repeated-measure ANOVAs were executed on STATA 12 (STATA Corp., College Station, TX). A *p* value of < 0.05 was considered statistically significant with *p* < 0.05 (*), *p* < 0.01 (**), *p* < 0.001 (***), and *p* < 0.0001 (****).

## Results

### Adjuvant metformin reduces inflammatory cytokine levels and nitric oxide production in vitro

**Fig. 1:**
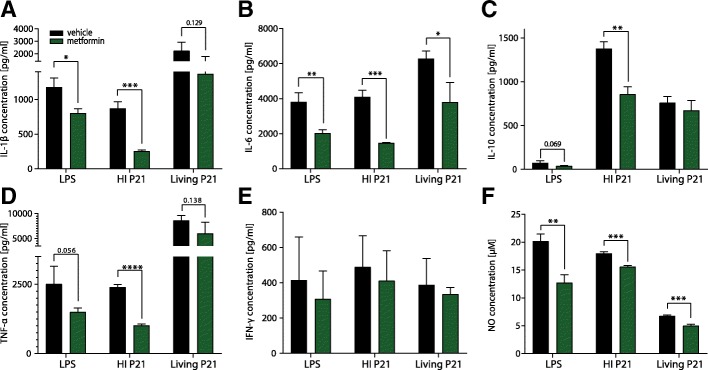
Reduced levels of inflammatory cytokines and NO after stimulation of astroglial cells in vitro. Primary neonatal rat astroglial cells were either stimulated with LPS, heat-inactivated (HI P21), or living *S*. *pneumoniae* serotype 3 (Living P21) in the presence or absence of metformin. Metformin significantly reduced levels of IL-1β (*p* = 0.0109 in LPS- and *p* = 0.0004 in HI P21 stimulated cells, **a**). IL-6 (*p* = 0.0050 in LPS-, *p* = 0.0003 in HI P21- and *p* = 0.0229 in living P21-stimulated cells, **b**) and nitric oxide (*p* = 0.0024 in LPS-, *p* = 0.0003 in HIP21- and p=0.0005 in living P21-stimualted cells, **f**) levels were significantly reduced by metformin independent of stimulating agents. IL-10 (*p* = 0.0015, **c**) and TNF-α (*p* < 0.0001, **d**) levels were significantly reduced in astroglial cells stimulated by HI P21 in the presence of metformin. No effect of metformin treatment on IFN-γ levels (**e**) was found

Primary astroglial cell cultures from infant rats were stimulated with LPS, heat-inactivated (HI P21), or living *S*. *pneumoniae* serotype 3 (living P21) and treated with metformin. All three stimuli potently activated astroglial cells in vitro as determined by increased levels of inflammatory cytokines and NO in the cell culture medium (Fig. 1a–f). In LPS-stimulated cells, metformin significantly reduced the release of IL-1β (*p* = 0.0109), IL-6 (*p* = 0.0050), and NO (*p* = 0.0024) and showed trends toward reduced levels of IL-10 (*p* = 0.069) and TNF-α (*p* = 0.056). Metformin also drastically reduced the release of IL-1β (*p* = 0.0004), IL-6 (*p* = 0.0003), IL-10 (*p* = 0.0015), TNF-α (*p* < 0.0001), and NO (*p* = 0.0003) induced by HI P21 stimulation. After exposure to living bacteria, adjuvant metformin to ceftriaxone treatment attenuated the levels of IL-6 (*p* = 0.0229) and NO (*p* = 0.0005), without significantly affecting the other measured cytokines. Of note, levels of IL-1β and TNF-α were considerably higher in astroglial cells exposed to living bacteria compared to HI P21 and LPS. Additionally, in contrast to the clear release of IL-10 in HI P21 and living P21 stimulated cells, LPS only marginally induced IL-10 production (Fig. 1c). In non-stimulated control cells, the production of cytokines and NO was at very low levels (IL-1β < 10 pg/ml; IL-6 < 105 pg/ml; IL-10 < 35 pg/ml; TNF-α < 65 pg/ml; IFN-γ < 85 pg/ml; NO < 0.5 μM, data not shown). The anti-inflammatory effect of metformin was further confirmed to be independent of serotype-specific surface polysaccharides, as metformin treatment also significantly reduced the release of IL-1β, IL-6, TNF-α, and NO after stimulation of astroglial cells with *S*. *pneumoniae* serotype 2 (D39, see Additional file 1). These anti-inflammatory properties were further documented to be capsule-independent as metformin significantly reduced the release of IL-1β, IL-6, TNF-α, and NO to a similar extent after astroglial cell stimulation with R6—a non-encapsulated mutant of D39 (see Additional file 1).

### Adjuvant metformin does not affect clinical parameters during acute PM

Overall, 174 infant Wistar rats were included in the study. All animals infected with *S*. *pneumoniae* serotype 3 (*n* = 140) developed meningitis, which was proven by growth of bacteria (≥ 10^6^ CFU/ml) in CSF samples obtained at 18 hpi and the appearance of meningitis symptoms reflected by clinical score < 5, weight loss, and changes in posture. Sixty-one infected infant rats were treated with adjuvant metformin (50 mg/kg) in addition to CRO, and 79 infected rats received CRO monotherapy. The slightly lower number of animals receiving metformin is explained by an initially performed dose-finding study evaluating different metformin concentrations, whereupon all animals receiving a metformin dose different from 50 mg/kg were excluded from subsequent analysis.

**Fig. 2:**
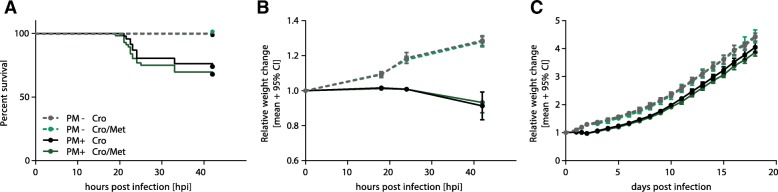
Survival and weight change in infant rats with pneumococcal meningitis. Infected rats showed significantly reduced survival compared to mock-infected animals, independent of treatment regimen (**a**). Weight change during acute meningitis (**b**) and long-term recovery (**c**) was significantly affected by infection but not by adjuvant metformin therapy

Survival was significantly reduced in infected animals (PM+) compared to mock-infected animals (PM−). Adjuvant metformin treatment did not affect survival in neither infected nor non-infected animals compared to animals treated with CRO monotherapy (Fig. 2a). Relative weight change showed a significant difference between infected and mock-infected animals during acute infection. In both infection groups, however, relative weight change was not altered by adjuvant metformin treatment neither during acute phase nor by chronic application (Fig. 2b, c).

### Adjuvant metformin therapy reduces levels of inflammatory cytokine in CSF and cortical necrosis but not hippocampal apoptosis

**Fig. 3:**
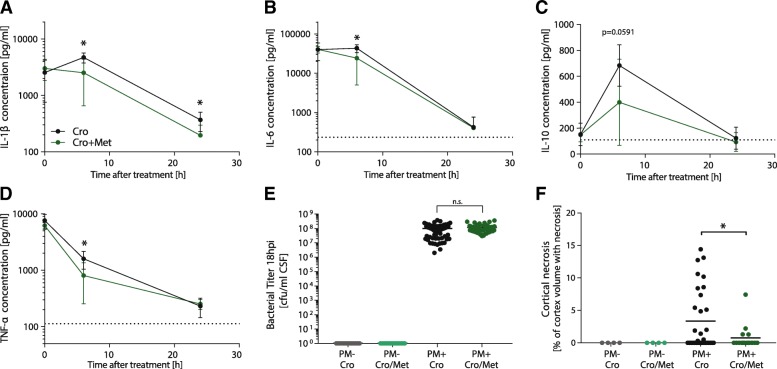
Inflammatory CSF cytokine levels, bacterial titers and cerebral complications during acute pneumococcal meningitis. Six hours after treatment initiation, adjuvant metformin significantly reduced levels of IL-1β (*p* = 0.015 and *p* = 0.034 at 24 h after treatment initiation, **a**), IL-6 (*p* = 0.036, **b**), and TNF-α (*p* = 0.044, **d**) and showed a trend toward reduced IL-10 levels (*p* = 0.059, **c**), despite having comparable CSF cytokine levels and bacterial titers at time of therapy initiation (**a**–**e**). Adjuvant metformin significantly reduced levels of cortical necrosis at 42 h post infection (*p* = 0.0101, **f**)

Inflammatory cytokines were measured before treatment initiation and at 6 h and 24 h after therapy start, representing 18, 24, and 42 hpi. Immediately before treatment initiation with CRO ± metformin at 18 hpi, infected animals from both groups—adjunctive metformin and CRO monotherapy—showed comparable levels of CSF cytokines and bacterial CSF titers, representing a comparable degree of infection and neuroinflammation at time of treatment initiation (Fig. 3a–e). Six hours after treatment initiation, adjuvant metformin therapy significantly reduced CSF levels of the inflammatory cytokines IL-1β (*p* = 0.015 and *p* = 0.034 at 24 h after treatment initiation), IL-6 (*p* = 0.036), and TNF-α (*p* = 0.044). No significant difference was found for IFN-γ and IL-10, although IL-10 showed a trend toward reduction (*p* = 0.059, Fig. 3c).

No cortical damage was found in mock-infected animals. Infected animals treated with adjuvant metformin had significantly reduced cortical necrosis compared to infected animals receiving CRO monotherapy (mean of damaged cortex volume 0.76% ± 0.47, *n* = 16, vs. 3.34% ± 0.83 in CRO monotherapy, *n* = 32, *p* = 0.0101, Fig. 3f). Apoptotic cells were found in the subgranular zone of the hippocampal dentate gyrus. Hippocampal apoptosis was found at physiological levels in mock-infected animals. Adjuvant metformin treatment did not significantly affect hippocampal apoptosis in infected animals (see Additional file 2).

### Metformin treatment reduces PM-induced hearing loss and protects spiral ganglion neurons

**Fig. 4:**
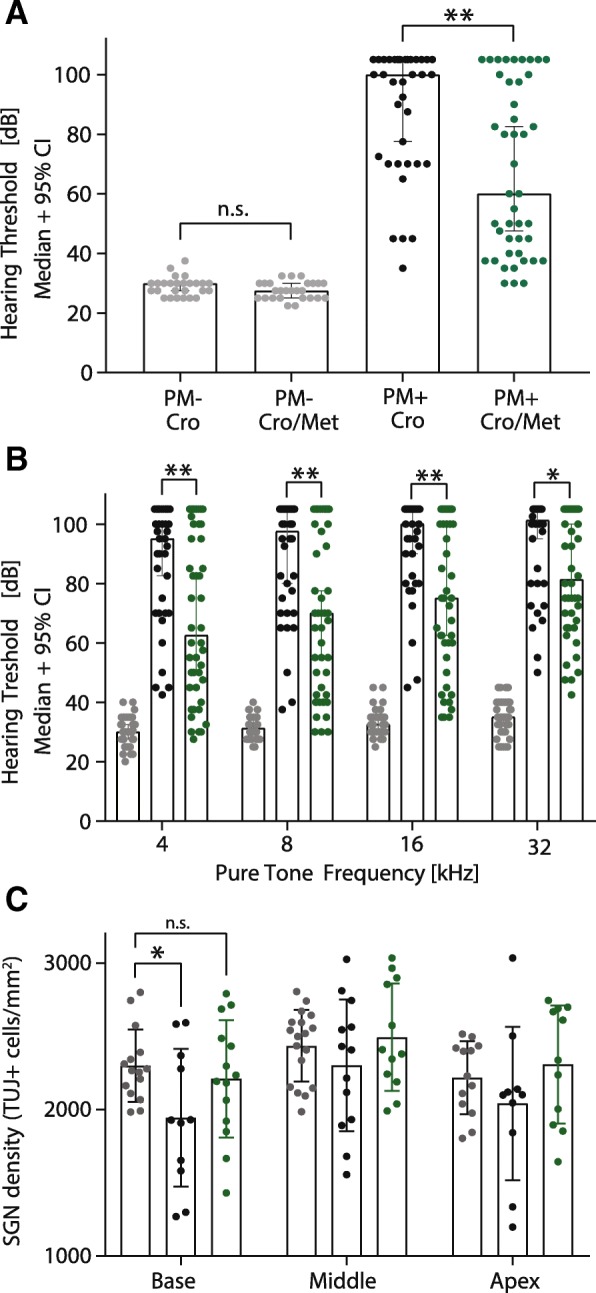
Hearing capacity and spiral ganglion neuron (SGN) density assessed 5 weeks after infection. Metformin therapy significantly improved click (*p* = 0.0015, **a**) and pure tone (4 kHz *p* = 0.0023, 8 kHz *p* = 0.0026, 16 kHz *p* = 0.0029, 32 kHz *p* = 0.0248, **b**) hearing thresholds in infected animals, without having an effect on hearing capacity in healthy animals (**a**). SGNs from the basal turn were protected from PM-induced cell death by metformin therapy (*p* = 0.0219, **c**)

Five weeks after infection, hearing thresholds were determined by recording response to click stimulations and pure tones on both ears. In infected animals, metformin treatment significantly improved click-induced hearing thresholds compared to animals with CRO monotherapy (60 dB, *n* = 44, in metformin therapy vs. 100 dB, *n* = 36 in CRO monotherapy, *p* = 0.0015, Fig. 4a). Pure tone ABR showed higher thresholds for all assessed frequencies (4, 8, 16, and 32 kHz) in infected animals compared to mock-infected animals (*n* = 44, *p* ≤ 0.0001, two-way ANOVA, Fig. 4b). Infected animals receiving adjuvant metformin treatment showed significantly better pure tone hearing capacity than animals treated with ceftriaxone monotherapy (*p* = 0.0013, Fig. 4b). Frequency-specific analysis revealed that at all measured frequencies, hearing capacity was significantly improved in infected animals receiving adjuvant metformin compared to CRO monotherapy (4 kHz *p* = 0.0023, 8 kHz *p* = 0.0026, 16 kHz *p* = 0.0029, 32 kHz *p* = 0.0248, Fig. 4b).

The densities of spiral ganglion neurons (SGNs) were assessed by counting SGNs in the basal, middle, and apical turns of mid-modiolar cochlea sections. A significant reduction of SGNs in the basal turn was determined in infected animals treated with CRO monotherapy compared to mock-infected animals (1945 ± 141.7, SGNs/mm^2^ in CRO monotherapy animals, *n* = 11 vs. 2299 ± 63.6 SGNs/mm^2^ in mock-infected animals, *n* = 15, *p* = 0.0197). This PM-induced neurotoxicity of SGNs was prevented by adjuvant metformin treatment with no detectable SGN loss in these animals (2211 ± 107.1 SGNs/mm^2^ in animals receiving metformin, *n* = 14, *p* = 0.475, Fig. 4c). A repeated-measure ANOVA, which considered the different cochlear turns as repeated measurements, revealed a significant protective effect of adjuvant metformin on SGN density over all cochlear turns in infected rats compared to CRO monotherapy (*p* = 0.0219).

### Metformin does not promote neurogenesis in the hippocampal neural stem cell niche

**Fig. 5:**
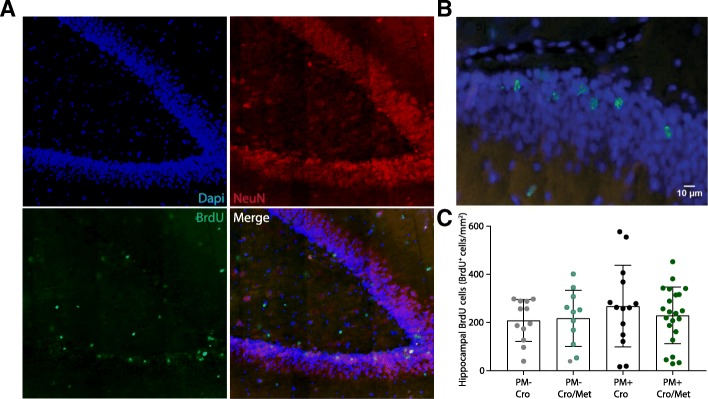
Quantification of BrdU^+^ cells in the NeuN^+^ region of the hippocampal dentate gyrus. BrdU-positive neurons (green) are clearly visible in the granular zone of the dentate gyrus (**a**, **b**). Adjuvant metformin treatment did not promote neurogenesis neither after experimental PM nor in mock-infected animals, as no significant differences in BrdU^+^ cell counts was detected between animals receiving metformin treatment and animals receiving CRO monotherapy (**c**)

Neurogenesis was defined by cells being BrdU^+^ and NeuN^+^, i.e., cells which have proliferated and incorporated BrdU after acute infection and successfully matured to neurons and integrated into existing hippocampal network 5 weeks after infection. BrdU^+^ cells were mostly found in the subgranular zone and the granule cell layer of the dentate gyrus (DG) (Fig. 5a, b), indicating their integration into hippocampal network. BrdU^+^ cells in the NeuN^+^ region of the DG were quantified to assess neuronal proliferation and survival. The results revealed no significant difference between mock-infected and infected animals as well as animals which were chronically treated with metformin or their littermates which received chronic vehicle injection (Fig. 5c).

## Discussion

In the present study, we investigated the anti-inflammatory, neuroprotective, and neuroregenerative effects of adjuvant metformin in PM. Our results demonstrate that treatment with adjuvant metformin in pediatric experimental PM decreases neuroinflammation, as reflected by reduced levels of inflammatory cytokines in the CSF, resulting in an attenuated cortical necrosis. These anti-inflammatory properties of metformin were further supported by results from additional in vitro experiments. Furthermore, metformin protected SGNs from PM-induced cell death and reduced PM-induced hearing loss.

Damage to the brain caused by PM is multifactorial, but mainly associated with an excessive inflammation, resulting in long-lasting neurological sequelae [5, 29, 33]. Rapid growth of *S*. *pneumoniae* in the subarachnoid space and released bacterial products induce a severe inflammatory response. This response may even be exacerbated after treatment with bacteriolytic antibiotics [4]. Based on these observations, adjunctive therapies are needed to attenuate the hyperinflammation in the brain during PM. Dexamethasone—an anti-inflammatory corticosteroid—is currently recommended as adjuvant therapy for adults and children with PM [38]. We have previously shown in the present model that therapy with dexamethasone showed only limited protection from PM-induced hearing loss and aggravated mortality, weight loss, and hippocampal injury with associated learning deficiency when compared to CRO monotherapy [32, 40, 61]. In the present study, we specifically compared metformin therapy to CRO monotherapy but not to adjuvant dexamethasone therapy.

In vitro, primary neonatal rat astroglial cells were stimulated with LPS, heat-inactivated and living *S*. *pneumoniae* in the presence or absence of metformin. The anti-inflammatory effect of metformin in LPS-stimulated macrophages and microglia has already been shown in vitro, where metformin significantly reduced the release of inflammatory cytokines and NO in an AMPK-dependent manner [44, 50]. The present in vitro results confirm the anti-inflammatory effects of metformin in LPS-induced neuroinflammation in primary astroglial cells. Furthermore, metformin reduced cytokines and NO levels caused by stimuli relevant for PM-induced neuroinflammation, like heat-inactivated or living pneumococci (Fig. 1a–f). We only found statistical trends for reduced IL-1β and TNF-α levels after stimulation with living P21 and treatment with metformin in vitro. However, the anti-inflammatory effect of metformin was further confirmed when stimulating the cells with living D39 and R6 with significant reduction for IL-1β, IL-6, TNF-α, and NO. Significant reduction of IL-1 β, IL-6, and TNF-α were also observed in the CSF of infected animals treated with metformin. As cytokines are important mediators of cerebral damage during PM, any reduction in inflammatory mediators might mediate neuroprotection in vivo [4]. The induction of most inflammatory mediators was comparable between the different stimuli, but we found notable differences such as poorly induced IL-10 levels after stimulation with LPS (Fig. 1c) or significantly higher IL-1β and TNF-α responses of cells exposed to living pneumococci (Fig. 1a, d). Lower levels of IL-1β and TNF-α in heat-inactivated pneumococci might be explained by the heat-lability of the highly immunogenic pneumolysin [13, 62]. Notably, the anti-inflammatory effect of metformin in vitro was confirmed after stimulation with a non-vaccine pneumococcal serotype (serotype 2, D39) and its non-encapsulated mutant R6, demonstrating that metformin’s anti-inflammatory properties are serotype- and capsule-independent and also potently reduces inflammatory mediators after infection with a non-vaccine serotype (Additional file 1). Low and unaffected levels of IFN-γ can be explained by the absence of T lymphocytes and NK cells in our cell culture, the two cell types mainly responsible to induce IFN-γ responses [63]. Additionally, IFN-γ serves mostly to combat intracellular pathogens [63], and only to a lesser extend against extra-cellular bacteria like *S*. *pneumoniae*. Comparison between the different stimuli should be interpreted with caution, since the kinetics of cytokine release may be different for each stimulus and we determined the accumulation of cytokines in the supernatant 24 h after stimulation.

In the in vivo study, the dose of metformin (50 mg/kg) was chosen based on preliminary pilot study and other independent studies [43, 51]. The use of a higher metformin dose (200 mg/kg) in our pilot study resulted in significantly increased mortality and hippocampal apoptosis in infected animals treated with adjuvant metformin compared to infected animals treated with CRO monotherapy (see Additional file 2). On the other hand, 20 mg/kg metformin did not protect from PM-induced cortical damage (data not shown). Although adjuvant metformin has been reported to cause weight loss in type 2 diabetes patients [64], we did not detect significant differences in weight change between animals receiving adjuvant metformin and animals with CRO monotherapy during acute (Fig. 2b) and post-acute (Fig. 2c) phases of PM—independent of infection with *S*. *pneumoniae*. In addition, metformin treatment did not affect survival (Fig. 2a). Together, these data indicate that 50 mg/kg metformin during acute PM and during recovery from PM is safe as it does not affect mortality or weight loss. Although we found promising results using 50 mg/kg metformin as adjuvant therapy in PM, further studies are needed to identify the influence of dosing on these reported effects.

Inflammatory cytokines produced by infiltrating leukocytes and brain-resident cells are crucially involved in the pathogenesis of PM [4]. During the acute phase of PM, levels of pro-inflammatory cytokines are drastically increased [4, 22, 55]. Metformin-dependent AMPK activation was shown to induce anti-inflammatory effects [43]. In this study, we found that adjuvant metformin significantly reduced CSF levels of inflammatory cytokines in vivo (Fig. 3) and in vitro (Fig. 1). In vivo, treatment with CRO monotherapy caused a transient increase of IL-1β and IL-6. This may be due to the antibiotic-induced lysis of bacteria caused by CRO—inducing a brisk release of highly immunogenic bacterial products [65]—leading to an elevated acute inflammation [4, 66, 67]. In contrast, levels of IL-1β and IL-6 decreased in animals treated with adjuvant metformin, indicating its anti-inflammatory effect. The kinetic of TNF-α release was different, with a steady decrease in infected animals, independent of the treatment. However, the decrease was more pronounced in animals treated with adjuvant metformin 6 h after therapy. No significant reduction of IFN-γ by adjuvant metformin could be detected in vivo and in vitro. For IL-10, only a trend toward reduction when treated with adjuvant metformin was found in vivo (*p* = 0.0591, Fig. 3c). Our findings of reduced neuroinflammatory parameters in vitro and in vivo are in line with previously reported studies, where metformin was able to reduce neuroinflammation in different experimental neuroinflammatory diseases [43–45, 50].

The pathogenesis of cortical brain injury is complex and involves vasculitis in cerebral blood vessels caused by inflammation of the surrounding subarachnoid space [24, 25, 55, 68]. With adjuvant metformin therapy, a significant reduction in cortical necrosis was detected (Fig. 3f). We hypothesize that an overall reduction of inflammatory parameters (cytokines and NO) by adjuvant metformin within the first hours after treatment initiation led to an attenuation of cortical damage—a correlation previously found with other anti-inflammatory adjuvant therapies in this infant rat PM model [58, 60, 69]. Comparable CSF cytokine levels 24 h after treatment start between metformin-treated and untreated animals are explained by the fast decline of CSF cytokines after treatment initiation, leading to low or even undetectable levels at this time point. This is in line with previously reported clinical and experimental data [21, 58, 60, 69, 70]. Nevertheless, hippocampal apoptosis was not affected with this dose of adjuvant metformin (see Additional file 2). Notably, prevention of hippocampal apoptosis in experimental infant rat PM has so far only been repetitively described with caspase-3 inhibitors, inhibitors of matrix-metalloproteinase, and TNF-α converting enzyme (TACE) or modulators of neurogenesis [55, 58, 71–73].

During the acute phase of PM, infiltration of pneumococci and leukocytes from the CSF into the basal turn of the cochlea via the cochlear aqueduct causes damage to the sensory hair cells and spiral ganglion neurons, resulting in sensorineural hearing loss [3, 33, 35–37, 74]. Previous studies demonstrated the ototoxic effect of TNF-α in vivo and in vitro [75, 76], and reported a positive correlation of CSF TNF-α levels with increased hearing loss after PM [35]. Additionally, reactive oxygen species and NO are known mediators of blood-labyrinth barrier disruption and sensorineural hearing loss after bacterial meningitis [77–79]. A reduction of inflammatory cytokines during acute experimental PM was shown to improve subsequent hearing capacity [60, 69, 80]. In the present study, adjuvant metformin therapy significantly improved hearing capacity 5 weeks after infection (Fig. 4a–c). In infected animals, metformin treatment significantly improved median click hearing thresholds compared to animals with CRO monotherapy by 40 dB (60 dB vs. 100 dB in CRO monotherapy, *p* = 0.0015, Fig. 4a). Frequency-dependent analysis showed that hearing was significantly improved by adjuvant metformin treatment in all assessed frequencies (4, 8, 16, and 32 kHz, Fig. 4b). In previous work, Perny et al. demonstrated that SGNs were affected depending on the severity of infection. The highest degree of SGN loss occurred in a basal to apical gradient, with a more pronounced hearing loss for the higher frequencies [35]. In accordance with these data, quantification of SGNs presented a significant reduction of SGNs in the basal turn of infected animals treated with CRO monotherapy, an effect that was prevented by adjuvant metformin therapy (Fig. 4d). Protection of SGNs during acute PM is crucial to maintain hearing capacity [35]. Previous studies using otoprotective compounds that attenuated the loss of SGNs were able to decrease PM-induced sensorineural hearing loss [60, 80]. Repeated-measure ANOVA showed a significant protective effect of adjuvant metformin in infected animals over all three cochlear turns, explaining the improved hearing threshold in all assessed frequencies. Thus, we suggest that metformin also reduced the levels of ototoxic inflammatory mediators in the cochlea and protected the SGNs from PM-induced toxicity. However, a significant protection of SGNs was only demonstrated in the basal turn, representing high-frequency hearing. Therefore, there must be additional explanations for the improvement of hearing capacity at middle and low frequencies. Since metformin is known to protect auditory hair cells in an ototoxicity model mediated by gentamicin [48], a preservation of cochlear hair cells during PM might explain our reported improvement of pure tone hearing capacity at all measured frequencies. The protection of presynaptic ribbons of surviving hair cells would be another possible explanation for improved hearing without detectable changes in SGNs [35].

Neurogenesis describes the generation, maturation, and integration of newborn neurons into the pre-existing neuronal network [81]. It has recently been shown that metformin promotes neurogenesis and neuronal differentiation in the hippocampus and thereby improves spatial memory formation in rodents [42, 49, 82]. In this study, adjuvant metformin did not significantly increase the number of surviving BrdU^+^ cells in the NeuN^+^ region of the DG—neither in infected nor in mock-infected animals. A possible explanation might be the developmental stage of the hippocampus. Most studies showing significantly increased neurogenesis upon metformin treatment were performed in adult or adolescent models [49, 50, 52, 53, 82]. These results might therefore differ from our infant model, in which the level of endogenous neurogenesis is already considerably high. In addition, studies that showed a significant increase in neurogenesis with increased numbers of BrdU^+^ cells in the hippocampal DG used a higher dose of metformin (200 mg/kg) [49, 82]. In our study, we had to reduce the dose to 50 mg/kg, as 200 mg/kg metformin increased mortality during acute PM (see Additional file 2). This reduced dose might have limited the neuroregenerative capacity of metformin. Many studies reported increased neurogenesis after different CNS damage like traumatic brain injury [83], stroke [84], Alzheimer’s disease [85], or neonatal inflammatory pain [86]. We have previously shown in the present model that the rate of cell division in the dentate gyrus increased significantly in the first days after infection [87]. However, no statistical difference between infected and mock-infected animals was found when the survival of this pool of cells born shortly after infection was tested 5 weeks later. Nevertheless, this is in line with similar findings in a mouse model of PM showing a transient increase of neurogenesis after PM, which is not detectable anymore at 5 weeks after infection [88]—representing the time when we sacrificed our animals.

Generally, manipulation of the hippocampal neurogenic niche with the intention to induce and activate endogenous neuroregeneration after pneumococcal meningitis-induced cell death aims for compensating damage that occurred during acute infection and represents another alternative therapeutic approach to improve the outcome after pediatric pneumococcal meningitis. Therapies promoting endogenous neuronal repair by activating neural stem cells would allow a therapeutic window that could be initiated later and for longer time when compared to adjunctive anti-inflammatory substances given during acute pneumococcal meningitis. It was therefore recently argued that future research should focus increasingly on neuroregeneration [89]. However, different neurogenic substances—experimentally shown to induce neurogenesis and improve learning and memory in non-infectious models [49, 72, 82]—failed to increase neurogenesis after pneumococcal meningitis [72]. Furthermore, for other neurogenic substances like neurotrophin-3 and BDNF, it has not been investigated so far whether the improved outcomes derive from neuroprotection during acute infection or increased neuroproliferation during chronic application [71, 90–92]. Nevertheless, the combination of anti-inflammatory and neuroprotective metformin with a potent inducer of neuroregeneration (BDNF or DHF [93]) may potentially improve the chance for successful neuroregeneration after PM-induced hippocampal damage.

## Conclusion

Treatment with metformin is neuro- and otoprotective in experimental pediatric PM. Metformin exerts its neuroprotective effects by reducing neuroinflammation, demonstrated by decreased CSF levels of inflammatory cytokines and reduced NO production in vitro and in vivo with subsequently reduced cortical damage. In addition, adjuvant metformin improves hearing capacity compared to standard ceftriaxone monotherapy and protected spiral ganglion neurons from PM-induced cell death. Although we could not confirm metformin’s neuroregenerative properties in this study, the results identify metformin as a highly promising therapeutic option to improve the outcome of PM and other neuroinflammatory diseases.

## Additional files

Additional file 1: Inflammatory mediators after *S. pneumoniae* serotype 2 infection in vitro. Primary neonatal rat astroglial cells were either stimulated with living *S. pneumoniae* serotype 2 (D39) or its non-encapsulated mutant R6 in presence or absence of metformin. Metformin significantly reduced levels of IL-1β (**A**, both *p* < 0.0001), IL-6 (**B**, *p* < 0.0001 for D39 and *p* = 0.0006 for R6), TNF-α (**D**, *p* < 0.0001 for D39 and *p* = 0.0002 for R6) and nitric oxide (**E**, both *p* < 0.0001). Levels of IL-10 were not affected by treatment with metformin (**C**). (PDF 376 kb)

Additional file 2: Survival, cortical necrosis and hippocamapal apoptosis in dose-fining study. Mortality was significantly increased by adjunctive 200 mg/kg metformin compared to CRO monotherapy and showed a trend to be higher than adjunctive 50 mg/kg metformin (**A**). Adjunctive 200 mg/kg metformin significantly reduced cortical necrosis at 42 hpi (**B**), while showing significantly increased hippocampal apoptosis (**C**) compared to CRO monotherapy. (PDF 385 kb)

## Abbreviations

ABR: Auditory brainstem response
AMPK: Adenosine monophosphate-activated protein kinase
BHI: Brain heart infusion
BrdU: Bromodeoxyuridine
CFU: Colony-forming unit
CNS: Central nervous system
CRO: Ceftriaxone
CSBA: Columbia sheep blood agar
CSF: Cerebrospinal fluid
DAPI: 4′,6-Diamidino-2-phenylindole
DG: Dentate gyrus
HI: Heat-inactivated
IVC: Individually ventilated cages
LPS: Lipopolysaccharide
NO: Nitric oxide
P21: Clinical *S*. *pneumoniae* strain (serotype 3)
PFA: Paraformaldehyde
PM: Pneumococcal meningitis
PNS: Peripheral nervous system
RNS: Reactive nitrogen species
ROS: Reactive oxygen species
SGN: Spiral ganglion neurons
SPL: Sound pressure level
TACE: TNF-α converting enzyme
