# Supplementary figures and images for: Metformin mediates neuroprotection and attenuates hearing loss in experimental pneumococcal meningitis
Source: J Neuroinflammation. 2019 Jul 27;16:156. doi: 10.1186/s12974-019-1549-6 (PMC6660697; doi:10.1186/s12974-019-1549-6)

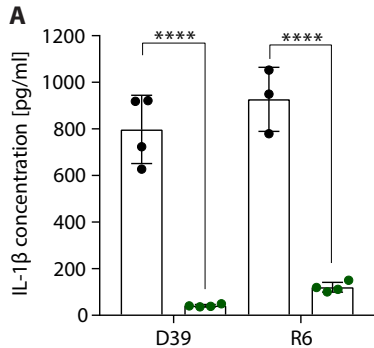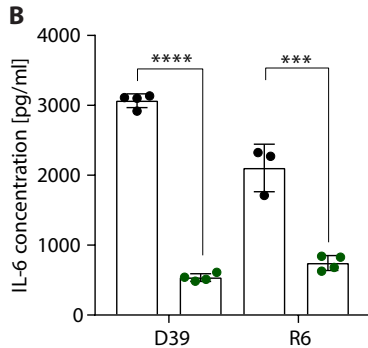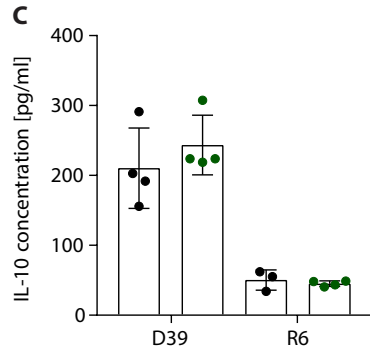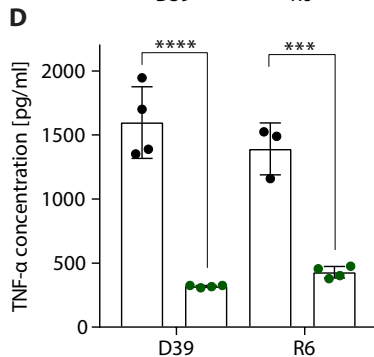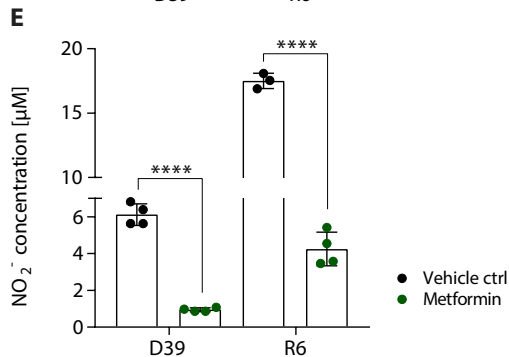

● Vehicle ctrl  
● Metformin

Supplement: Supplementary file 1 — Inflammatory mediators after S. pneumoniae serotype 2 infection in vitro. Primary neonatal rat astroglial cells were either stimulated with living S. pneumoniae serotype 2 (D39) or its non-encapsulated mutant R6 in presence or absence of metformin. Metformin significantly reduced levels of IL-1β (A, both p < 0.0001), IL-6 (B, p < 0.0001 for D39 and p = 0.0006 for R6), TNF-α (D, p < 0.0001 for D39 and p = 0.0002 for R6) and nitric oxide (E, both p < 0.0001). Levels of IL-10 were not affected by treatment with metformin (C). (PDF 376 kb) [file 12974_2019_1549_MOESM1_ESM.pdf]

**A**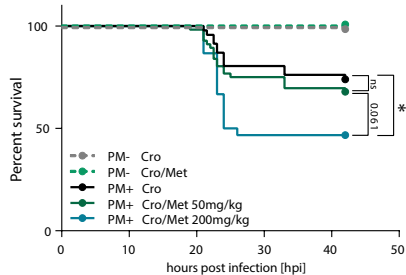**B**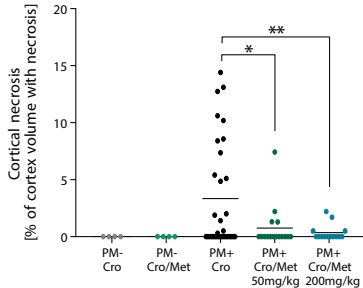**C**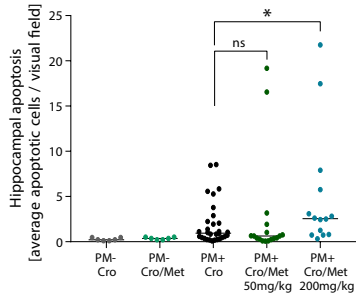

Supplement: Supplementary file 2 — Survival, cortical necrosis and hippocamapal apoptosis in dose-fining study. Mortality was significantly increased by adjunctive 200 mg/kg metformin compared to CRO monotherapy and showed a trend to be higher than adjunctive 50 mg/kg metformin (A). Adjunctive 200 mg/kg metformin significantly reduced cortical necrosis at 42 hpi (B), while showing significantly increased hippocampal apoptosis (C) compared to CRO monotherapy. (PDF 385 kb) [file 12974_2019_1549_MOESM2_ESM.pdf]
